# Impact of Associated Infections on the Clinical Course and Risk-Stratification Profile of Pulmonary Embolism: Comparative Analysis Using PESI, Wells, Padua, and IMPROVE Scores

**DOI:** 10.3390/diagnostics16172779

**Published:** 2026-08-29

**Authors:** Daniela Nicoleta Crisan, Raluca Dumache, Talida Georgiana Cut, Alexandra Herlo, Marius Florentin Popa, Lucian-Flavius Herlo, Nina Ivanovic, Andreea Nelson Twakor, Gabriela-Florentina Țapoș, Adelina Raluca Marinescu

**Affiliations:** 1 Doctoral School, “Victor Babes” University of Medicine and Pharmacy Timisoara, 300041 Timisoara, Romania; daniela.crisan@umft.ro (D.N.C.); flavius.herlo@umft.ro (L.-F.H.); nina.ivanovic@umft.ro (N.I.); 2 Department of Forensic Medicine, Bioethics, Medical Ethics and Medical Law, “Victor Babes” University of Medicine and Pharmacy Timisoara, 300041 Timisoara, Romania; raluca.dumache@umft.ro; 3 Center for Ethics in Human Genetic Identifications, “Victor Babes” University of Medicine and Pharmacy Timisoara, E. Murgu Square, Nr. 2, 300041 Timisoara, Romania; 4 Department XIII, Discipline of Infectious Diseases, “Victor Babes” University of Medicine and Pharmacy Timisoara, 2 Eftimie Murgu Square, 300041 Timisoara, Romaniaadelina.marinescu@umft.ro (A.R.M.); 5 Department of Forensic Medicine, “Sf. Apostol Andrei” Emergency County Hospital, 900439 Constanta, Romania; 6 Department of Dermatology, “Victor Babes” University of Medicine and Pharmacy Timisoara, E. Murgu Square, Nr. 2, 300041 Timisoara, Romania; 7 Department of Internal Medicine, “Sf. Apostol Andrei” Emergency County Hospital, 145 Tomis Blvd., 900591 Constanta, Romania; andreea.purcaru@365.univ-ovidius.ro (A.N.T.); gabriela.tapos@umft.ro (G.-F.Ț.)

**Keywords:** pulmonary embolism, infection-associated pulmonary embolism, COVID-19, venous thromboembolism, PESI score, WELLS score, PADUA score, IMPROVE, mortality, risk assessment

## Abstract

**Background:** Pulmonary embolism (PE) remains a major cause of cardiovascular morbidity and mortality, and associated infections may modify its clinical course. This study evaluated the impact of viral infections on pulmonary embolism severity, mortality, and the performance of established risk scores. **Methods:** This retrospective observational study included 729 patients with confirmed PE admitted between January 2020 and December 2025. Patients were grouped according to the presence of associated infections. We analysed clinical data, comorbidities, thrombus localization, laboratory parameters, infection type, treatment, mortality, and Pulmonary Embolism Severity Index (PESI), Wells, Padua, and International Medical Prevention Registry on Venous Thromboembolism (IMPROVE) scores. We performed between-group comparisons, logistic regression, and ROC curve analyses. **Results:** Patients with associated infections had higher crude mortality than those without infections (27.7% vs. 18.4%, *p* = 0.006). They also showed higher PESI and IMPROVE scores for venous thromboembolism (VTE), increased white blood cell (WBC) count, markedly elevated D-dimers, longer prothrombin time, and different thrombus localization. Viral infection category was independently associated with mortality, with the IMPROVE score showing the highest ROC discrimination among evaluated scores. **Conclusions:** Associated infections define a higher-risk PE phenotype. Risk stratification should integrate infection status, laboratory markers, and conventional PE/VTE scores.

## 1. Introduction

Pulmonary embolism (PE) remains a frequent emergency and inpatient condition requiring integrated assessment of diagnostic probability, short-term severity, anticoagulation strategy, and disposition, with contemporary management emphasizing avoidance of both underdiagnosis and overtreatment [1]. It remains a major global cardiovascular emergency, with an estimated annual incidence of approximately 39–115 cases per 100,000 individuals and reported mortality declining from historical estimates of 18–30% to more recent PE-related mortality estimates of 1–3% in contemporary cohorts [2]. A recent World Health Organization Mortality Database analysis including data from 73 countries showed that the global age-standardized PE-related mortality rate decreased from 3.49 per 100,000 in 2001 to 2.42 per 100,000 in 2023, although important disparities persisted [3]. Mortality decreased in high-income countries from 3.68 to 2.20 per 100,000, while lower-to-middle-income countries showed an increase from 0.92 to 4.82 per 100,000 over the same period, emphasizing that PE remains unequally distributed as a cause of death worldwide [3].

Current PE care is increasingly individualized according to hemodynamic status, right ventricular dysfunction, bleeding risk, and the availability of anticoagulant, thrombolytic, catheter-directed, or surgical strategies [4]. Despite improvements in diagnosis and treatment, PE remains an important contributor to global mortality, with persistent geographic and economic disparities in PE-related deaths [5]. Therefore, early risk stratification is essential to distinguish high-risk PE requiring urgent reperfusion from normotensive patients who need structured assessment for deterioration, bleeding, hospitalization, or outpatient care [6].

Several clinical scores are used in this context. The Pulmonary Embolism Severity Index (PESI) estimates mortality risk after PE using demographic, comorbidity, and clinical variables, and original PESI has shown stronger long-term mortality prediction than simplified PESI or revised Geneva scoring in one 10-year follow-up study [7]. The Wells score estimates pretest probability of PE and, when combined with age-adjusted D-dimer, may support safe PE exclusion in selected inpatients [8]. The Padua Prediction Score evaluates VTE risk in hospitalized medical patients, although obligatory scoring increased prophylaxis use without reducing VTE or mortality in a large retrospective cohort [9]. The International Medical Prevention Registry on Venous Thromboembolism (IMPROVE) score is another hospitalization-oriented VTE risk assessment model, aligning with current efforts to standardize VTE assessment, reassessment, prophylaxis, and electronic decision support [10].

Emerging evidence indicates that acute viral respiratory infections, and COVID-19 in particular, are associated with a distinct, more severe pulmonary embolism phenotype characterized by peripheral (segmental/subsegmental) thrombus distribution, heightened systemic inflammation, and increased mortality compared with PE occurring in the absence of infection. However, the extent to which conventional PE and VTE risk scores retain their prognostic value in this infected subgroup, and which laboratory or clinical parameters add incremental discriminatory information, remains incompletely characterized. The aim of this study was to evaluate the impact of associated viral infections on the clinical course, laboratory profile, and in-hospital mortality of patients with confirmed pulmonary embolism, and to compare the discriminatory performance of the PESI, Wells, Padua, and IMPROVE scores in this population.

## 2. Materials and Methods

### 2.1. Study Design, Inclusion and Exclusion Criteria

This retrospective observational study included patients diagnosed with pulmonary embolism and admitted to Arad County Clinical Hospital, Romania, between January 2020 and December 2025. The final study cohort comprised 729 patients, divided into two groups according to the presence or absence of an associated, laboratory-confirmed viral infection: patients with pulmonary embolism without associated infection and patients with pulmonary embolism with an associated viral infection.

PE was confirmed by computed tomography pulmonary angiography, based on the identification of an intraluminal filling defect within the pulmonary arterial tree, including the main, lobar, segmental, or subsegmental pulmonary arteries. Imaging findings compatible with acute pulmonary embolism included partial or complete arterial obstruction, contrast surrounding the thrombus on short- or long-axis views, eccentric or mural filling defects, and complete vessel occlusion when present.

All consecutive patients fulfilling the diagnostic criteria during the study interval were considered eligible. Patients were excluded only when essential diagnostic, clinical, infection-related, or outcome data were unavailable. The study was conducted according to the Declaration of Helsinki and approved by the Ethics Committee of Victor Babes University of Medicine and Pharmacy Timisoara, Romania, approval no. 39, dated 3 March 2025.

The primary outcome of this study was in-hospital mortality, defined as death from any cause occurring during the index hospital admission for pulmonary embolism. No post-discharge follow-up data were available.

### 2.2. Data Collection and Infection Assessment

Clinical data were extracted from institutional electronic medical records and anonymized before analysis. Recorded variables included age, sex, thrombus localization, comorbidities, respiratory support, intensive care interventions, treatment strategy, and in-hospital outcome. Comorbidities included diabetes mellitus, cancer, obesity, pulmonary hypertension, and cardiac failure. Pulmonary hypertension was defined based on a documented diagnosis in the patient’s medical record, supported by echocardiographic findings when available. Right-heart catheterization was not systematically performed and was therefore not required for classification. Cancer was defined as active malignancy documented at the time of the index hospital admission, including patients with an established diagnosis of malignancy who were receiving active treatment or had ongoing active disease. Thrombus location was classified according to the most proximal (highest-hierarchy) vessel involved on imaging (main pulmonary artery > lobar > segmental > subsegmental > truncus pulmonalis), such that each patient was assigned to a single, mutually exclusive anatomical-level category. Bilateral involvement and saddle-embolus configuration were not systematically and separately coded in this dataset; cases exhibiting these patterns, along with isolated peripheral/distal branch involvement, were grouped under ‘other localizations’.

PE with associated viral infection was defined strictly on the basis of a positive multiplex respiratory PCR result obtained at the time of admission for pulmonary embolism. Testing was performed using the BioFire respiratory multiplex panel and included SARS-CoV-2, influenza A and B, respiratory syncytial virus, human metapneumovirus, and parainfluenza virus. Clinical presentation and routine laboratory findings were considered supportive information but were not independently used to define infection status or determine group assignment. Patients without a laboratory-confirmed viral infection were classified in the PE without associated infection group. Patients with clinical and/or microbiological evidence of concomitant bacterial infection or bacterial superinfection were excluded from the study, as the present analysis was specifically designed to evaluate pulmonary embolism associated with laboratory-confirmed viral respiratory infections.

Laboratory values recorded within the first 24 h of admission included white blood cell count (×10^3^/µL), neutrophils (×10^3^/µL), lymphocytes (×10^3^/µL), platelet count (×10^3^/µL), creatinine (mg/dL), bilirubin (mg/dL), aspartate aminotransferase (AST, U/L), alanine aminotransferase (ALT, U/L), gamma-glutamyl transferase (GGT, U/L), glucose (mg/dL), sodium (mmol/L), potassium (mmol/L), procalcitonin (ng/mL), D-dimers (µg/mL FEU), prothrombin time (s), activated partial thromboplastin time (aPTT, s), international normalized ratio (INR), and total cholesterol (mg/dL). Treatment data included thrombolysis and anticoagulant therapy. Data on catheter-directed therapies, including endovascular thrombectomy, were not collected in this cohort.

### 2.3. Clinical Risk Scores

Pulmonary embolism risk scores were calculated using admission data for each patient. These included the PESI, Wells, Padua, and IMPROVE scores. Scores were analyzed in relation to infection status, and mortality. Although Wells and Padua were originally developed to estimate pre-test probability of PE and hospital-acquired VTE risk, respectively, rather than post-diagnosis mortality, they remain widely used in routine clinical assessment; their comparative performance was therefore evaluated to provide practically relevant information for clinicians applying these tools across the diagnostic-to-prognostic continuum.

### 2.4. Statistical Analysis

Statistical analyses were performed using IBM SPSS Statistics version 30.0 (IBM Corp., Armonk, NY, USA). Continuous variables were summarized as mean, standard deviation (SD), median, minimum, and maximum, while categorical variables were expressed as frequencies and percentages. Between-group comparisons were performed using independent-samples *t*-tests or non-parametric tests, as appropriate. Categorical variables were compared using chi-square or Fisher’s exact tests. Candidate predictors for the multivariable logistic regression model for in-hospital mortality were pre-specified a priori based on clinical and mechanistic relevance rather than derived from univariate screening, resulting in a final model of four variables: PESI score (a validated composite severity index), WBC count (a marker of the systemic inflammatory response), D-dimer (a marker of thrombotic burden), and any viral infection (the primary exposure of interest). All four predictors were entered simultaneously (Enter method); no stepwise selection or elimination was performed. None of the four variables had missing data, so the model was fitted on the full analytic cohort (*N* = 729; 152 in-hospital deaths; approximately 38 events per variable), and no imputation or case-wise exclusion was required. Multicollinearity among model predictors was assessed using tolerance and variance inflation factors (VIF); all values were below the conventional threshold of concern (VIF < 5; see footnote of the table in Section 3.5).

We additionally fitted a second multivariable logistic regression model incorporating the complete set of variables reaching *p* < 0.05 in univariable comparisons between survivors and non-survivors (age, pulmonary hypertension, WBC, neutrophil count, total bilirubin, AST, ALT, GGT, fasting blood glucose, procalcitonin, total cholesterol, D-dimer, prothrombin time, INR, and the Wells, Padua, IMPROVE, and PESI scores) together with viral infection status, all entered simultaneously (Enter method), with no variables excluded or eliminated.

Receiver operating characteristic (ROC) curve analysis evaluated the discriminatory performance of the final multivariable model, using the model-predicted probability of in-hospital death as the test variable, and of the PESI, Wells, Padua, and IMPROVE scores individually, using their raw score values. A *p*-value < 0.05 was considered statistically significant.

## 3. Results

### 3.1. Study Population and Grouping

The analyzed dataset comprised 729 patients with pulmonary embolism. Non-survivors were significantly older than survivors (71.39 ± 13.96 vs. 67.99 ± 13.95 years, t = −2.669, *p* = 0.008) and had higher PESI, Padua, IMPROVE, and Wells scores (all *p* ≤ 0.014), consistent with greater baseline severity in patients who died (Table 1). Among comorbidities, pulmonary hypertension was significantly less frequent in non-survivors (25.7% vs. 43.4%, χ^2^ = 15.087, *p* < 0.001), while diabetes, cancer, and cardiac failure did not differ between groups (all *p* > 0.4).

Thrombus localization showed a shift toward segmental involvement in non-survivors (43.0% vs. 30.2%), although the overall six-category comparison did not reach significance (χ^2^ = 9.844, df = 5, *p* = 0.080). Several inflammatory and organ-injury markers were markedly elevated in non-survivors, including WBC (13.28 ± 10.51 vs. 9.17 ± 4.81 × 10^3^/µL, U = 29,919.0, *p* < 0.001), neutrophils (U = 28,582.5, *p* < 0.001), procalcitonin (U = 30,859.0, *p* < 0.001), AST, ALT, GGT, total bilirubin, and glucose (all *p* ≤ 0.004), whereas lymphocytes, platelet count, creatinine, sodium, and potassium showed no significant differences. D-dimers were also significantly higher in non-survivors (20.01 ± 22.33 vs. 14.48 ± 18.24 µg/mL FEU, U = 50,078.0, *p* < 0.007), as was total cholesterol (199.94 ± 65.59 vs. 166.67 ± 45.61 mg/dL, t = −5.890, *p* < 0.001), and among coagulation parameters available for the PE-without-infection subgroup, prothrombin time and INR were prolonged in non-survivors (both *p* < 0.001) while aPTT was not (*p* = 0.443). Finally, any viral infection was significantly more common among non-survivors than survivors (34.9% vs. 23.9%, χ^2^ = 6.907, *p* = 0.009), supporting its role as an independent predictor of in-hospital mortality.

To further characterize the infection-associated phenotype, Table 2 and Table 3 additionally compare patients grouped by infection status.

According to the grouping variable used in the statistical output, 538 patients were classified in the PE group and 191 patients were classified in the PE with associated infections group (Table 2). The two groups were similar in age, with a mean age of 68.55 ± 14.22 years in the PE group and 69.12 ± 13.43 years in the PE with associated infections group. The median age was 70 years in the PE group and 71 years in the PE with associated infections group. Sex distribution was balanced in the overall cohort, with 372 male patients and 357 female patients. In the PE group, 265 patients were male and 273 were female, corresponding to 49.3% and 50.7%, respectively. In the PE with associated infections group, 107 patients were male and 84 were female, corresponding to 56.0% and 44.0%, respectively.

Thrombus localization differed between the two groups. In the PE group, thrombi were most frequently located in the lobar arteries, followed closely by the segmental arteries and main arteries. Specifically, lobar artery involvement was observed in 159 patients, segmental artery involvement in 156 patients, and main artery involvement in 138 patients. Subsegmental artery involvement was recorded in 20 patients, truncus pulmonalis involvement in 21 patients, and other localizations in 42 patients. In contrast, the PE with associated infections group showed a higher proportion of segmental artery involvement, which was present in 83 patients. Other localizations were recorded in 46 patients, lobar artery involvement in 41 patients, and main artery involvement in 20 patients. Subsegmental artery involvement was not recorded in the PE with associated infections group, while truncus pulmonalis involvement was identified in one patient.

The PE risk-score profile differed between groups. Mean PESI score was higher in the PE with associated infections group than in the PE group, whereas the Wells and Padua scores were lower. The mean PESI score was 110.00 ± 32.90 in the PE group and 123.41 ± 33.71 in the PE with associated infections group. The median PESI score was 110 in the PE group and 116 in the PE with associated infections group. The mean PESI class was 3 ± 1 in the PE group and 4 ± 1 in the PE with associated infections group. The Wells score was 1.83 ± 1.95 in the PE group and 1.16 ± 1.45 in the PE with associated infections group. The Padua score was 3.34 ± 2.34 in the PE group and 2.89 ± 2.02 in the PE with associated infections group. Conversely, the IMPROVE score was substantially higher in the PE with associated infections group, with a mean of 4.80 ± 1.68 compared with 2.62 ± 1.88 in the PE group.

Comorbidity distribution also differed according to the grouping variable. Pulmonary hypertension (PH) was recorded in 234 patients in the PE group and 55 patients in the PE with associated infections group, corresponding to 43.5% and 28.9%, respectively. Diabetes mellitus was present in 123 patients in the PE group and 54 patients in the PE with associated infections group. Cancer was recorded in 107 patients in the PE group and 28 patients in the PE with associated infections group. Obesity was recorded in 168 patients in the PE group and 73 patients in the PE with associated infections group, corresponding to 31.2% and 38.4%, respectively. Cardiac failure was highly prevalent in both groups, occurring in 354 of 538 valid PE cases (65.8%) and 126 of 190 valid PE with associated infections cases (66.3%); the association with group was not statistically significant (Pearson χ^2^ = 0.017, df = 1, *p* = 0.897).

In-hospital mortality also differed significantly between groups. Death occurred in 99 of 538 patients in the PE group (18.4%) compared with 53 of 191 patients in the PE with associated infections group (27.7%); the association was statistically significant (Pearson χ^2^ = 7.463, df = 1, *p* = 0.006), a finding also supported by Fisher’s exact test (two-sided *p* = 0.009).

**Figure 1:**
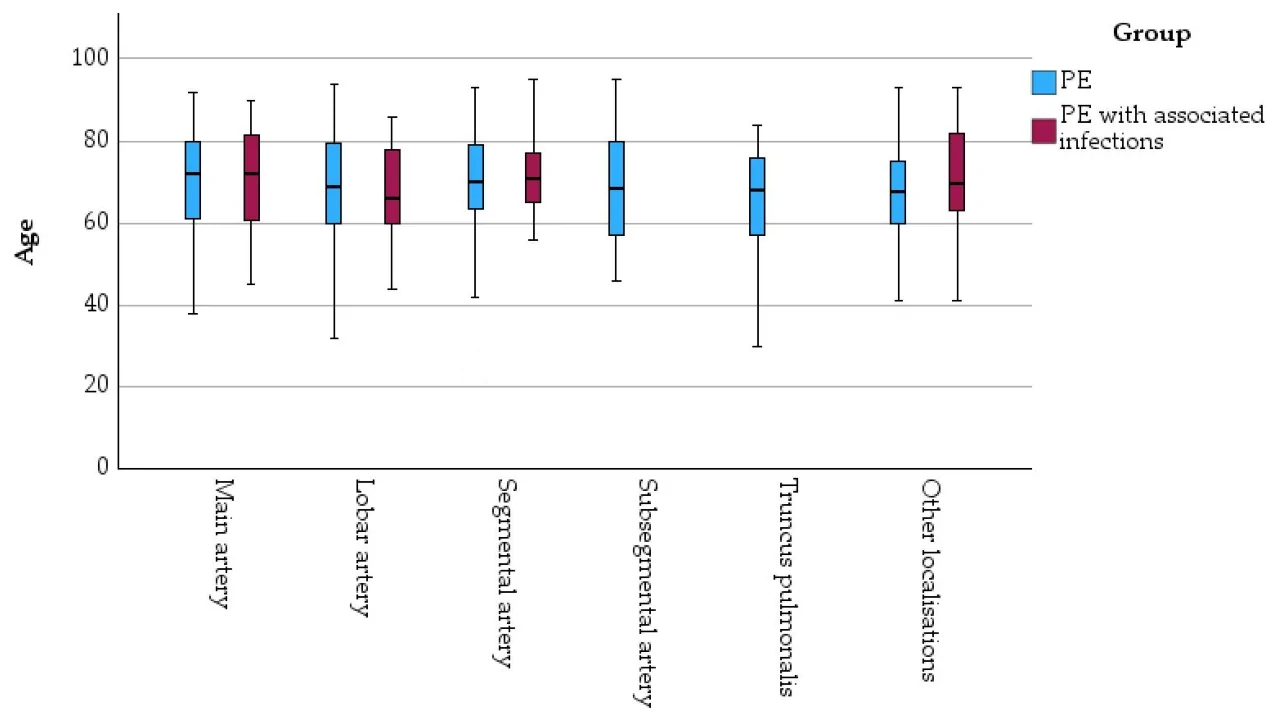
Age distribution by thrombus location according to group. *n* = 1 for truncus pulmonalis in the PE with associated infections group; box-and-whisker plot not generated for this category.

Age distribution was comparable across thrombus locations in both groups, with median values ranging from approximately 66 to 72 years and largely overlapping interquartile ranges (Figure 1).

The main, lobar, and segmental arteries showed similar median ages between the PE and PE with associated infections groups, while the widest age dispersion was observed in patients with truncus pulmonalis involvement in the PE group (range 30–84 years). Subsegmental artery and truncus pulmonalis involvement were rare in the PE with associated infections group.

**Figure 2:**
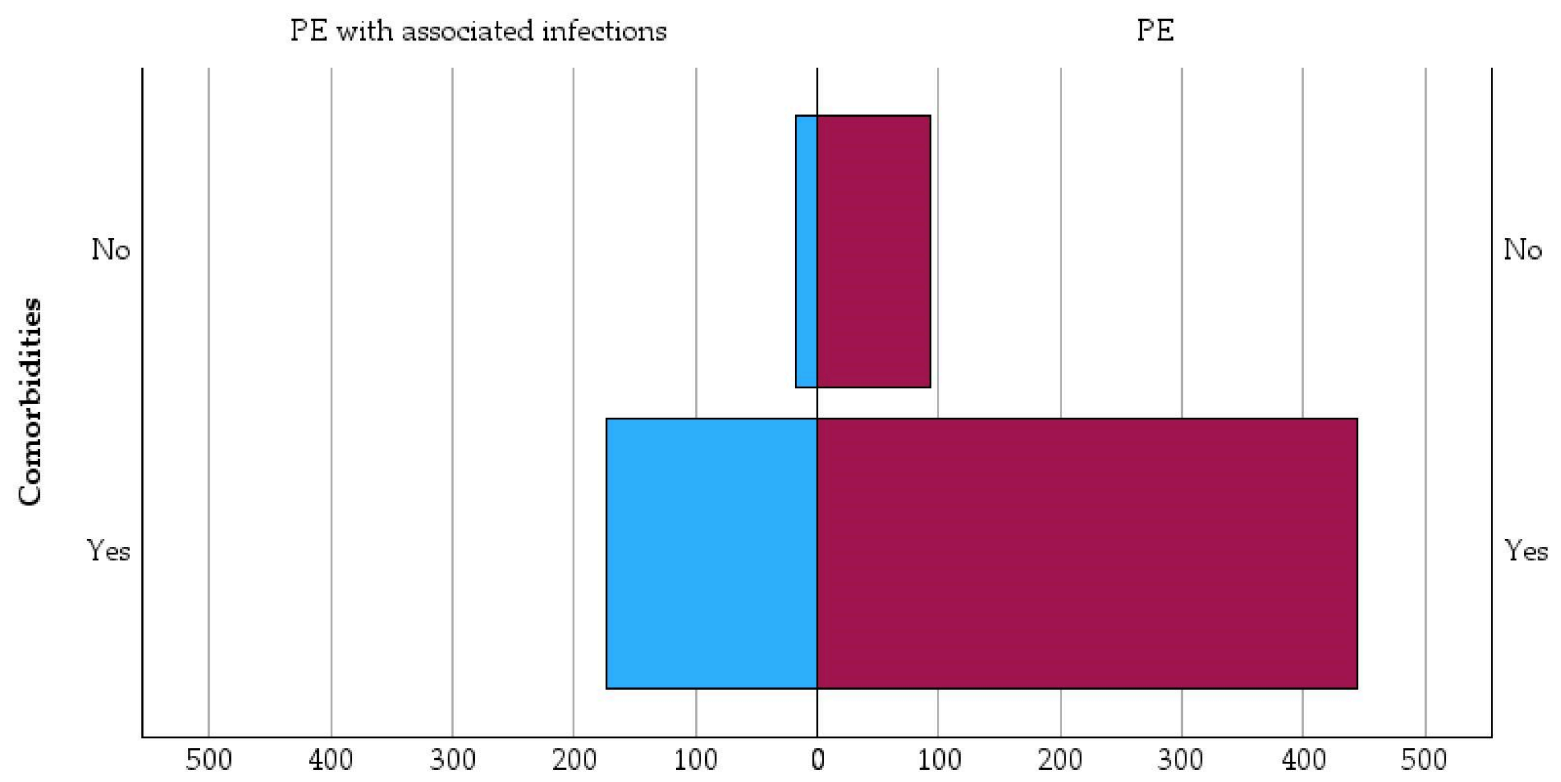
Distribution of comorbidity burden (presence vs. absence of comorbidities) in the PE and PE with associated infections groups.

Figure 2 shows the presence of comorbidities between the two study groups.

The large majority of patients in both groups had at least one recorded comorbidity, while only a small minority were free of comorbid conditions, and this pattern was similar between the PE and PE with associated infections groups.

**Figure 3:**
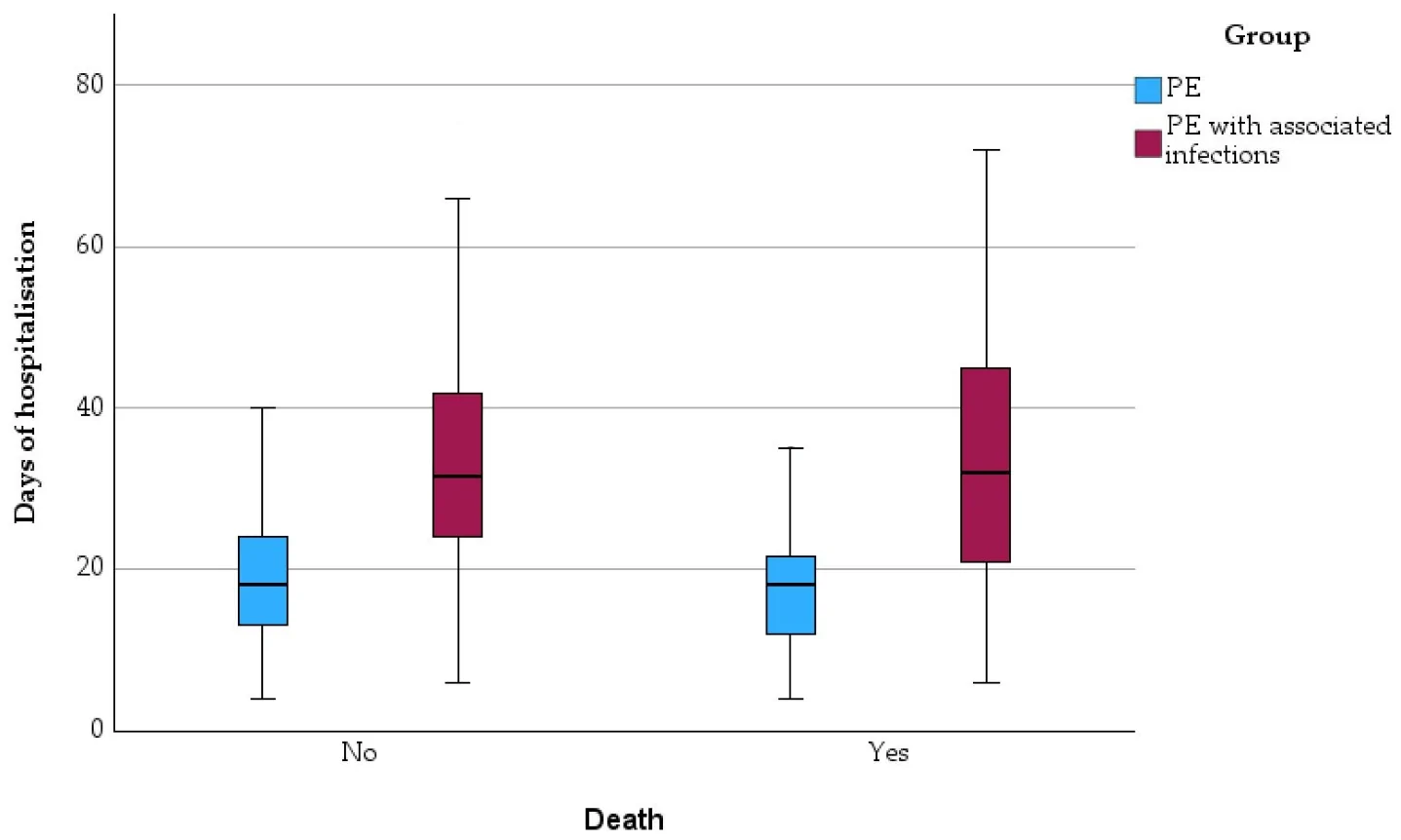
Length of hospital stay according to death outcome and group.

Figure 3 shows the distribution of hospitalization duration stratified by in-hospital death and group.

In both survivors and non-survivors, patients in the PE with associated infections group had a markedly longer hospital stay than those in the PE group, with median values of approximately 31–32 days compared with approximately 18 days in the PE group.

### 3.2. Distribution of Associated Infections

The viral infection profile showed a strong group association. Among patients in the PE group, no COVID-19, influenza A, influenza B, respiratory syncytial virus, human metapneumovirus, or parainfluenza cases were recorded. In the PE with associated infections group COVID-19 was the dominant viral infection, occurring in 172 patients, which represented 90.1% of the PE with associated infections group. Influenza B was recorded in seven patients, respiratory syncytial virus in five patients, influenza A in four patients, human metapneumovirus in two patients, and parainfluenza in one patient.

Within the infection group, defined exclusively by laboratory-confirmed viral infection, COVID-19 was the dominant pathogen, accounting for 172/191 (90.1%) of cases.

### 3.3. Laboratory Profile

The laboratory profile showed higher inflammatory and coagulation-related values in the PE with associated infections group for several parameters. Mean white blood cell count was 9.88 ± 5.49 in the PE group and 12.21 ± 5.61 in the PE with associated infections group, with medians of 10 and 11, respectively. D-dimer values were substantially higher in the PE with associated infections group, with a mean of 34.91 ± 24.05 and median of 27, compared with 8.79 ± 10.97 and median of 5 in the PE group. Prothrombin time was also higher in the PE with associated infections group, with a mean of 15.96 ± 6.51 and median of 14, compared with 14.29 ± 4.86 and median of 13 in the PE group.

Several biochemical markers showed smaller absolute differences between groups. Mean creatinine was 1.36 ± 4.06 in the PE group and 1.04 ± 0.51 in the PE with associated infections group, with medians of 1.06 and 0.90, respectively. Mean total bilirubin was 0.77 ± 0.78 in the PE group and 0.71 ± 0.89 in the PE with associated infections group. Mean AST and ALT values were numerically higher in the PE group, although the median values were higher in the PE with associated infections group. Mean cholesterol was higher in the PE with associated infections group, at 198 ± 50, compared with 165 ± 50 in the PE group. Sodium was lower in the PE with associated infections group, with a mean of 136 ± 4 compared with 139 ± 5 in the PE group.

The complete laboratory profile extracted from the custom tables is presented in Table 3.

### 3.4. Between-Group Comparisons of Continuous Variables

Independent-samples testing showed no statistically significant difference in age between groups. The mean age difference was −0.568 years for the PE group compared with the PE with associated infections group, with a two-sided *p*-value of 0.630 under the equal-variance assumption and 0.621 under the unequal-variance assumption. The corresponding Cohen’s d was −0.041, indicating a negligible standardized difference.

Risk scores differed significantly between groups. PESI score was significantly higher in the PE with associated infections group, with a mean difference of −13.406 when calculated as PE minus PE with associated infections. The two-sided *p*-value was <0.001, and the 95% confidence interval for the mean difference was −18.882 to −7.930 under the equal-variance assumption. The standardized effect size was moderate in magnitude, with Cohen’s d = −0.405. In contrast, Wells score was significantly lower in the PE with associated infections group, with a mean difference of 0.6702 for PE minus PE with associated infections, *p* < 0.001, and Cohen’s d = 0.365. Padua score was also lower in the PE with associated infections group, with a mean difference of 0.446, *p* = 0.019 under the equal-variance assumption and *p* = 0.012 under the unequal-variance assumption. The corresponding Cohen’s d was 0.198.

The largest between-group score difference was observed for the IMPROVE score. The PE with associated infections group had a substantially higher IMPROVE score than the PE group, with a mean difference of −2.179 for PE minus PE with associated infections. The difference was statistically significant, with *p* < 0.001. The 95% confidence interval for the mean difference was −2.482 to −1.876 under the equal-variance assumption and −2.467 to −1.891 under the unequal-variance assumption. Cohen’s d was −1.189, indicating a large standardized difference.

WBC count was also significantly higher in the PE with associated infections group, with a mean difference of −2.330, *p* < 0.001, and Cohen’s d = −0.422. D-dimers showed the strongest laboratory separation between groups, with a mean difference of −26.111, *p* < 0.001, and Cohen’s d = −1.686. Prothrombin time was significantly higher in the PE with associated infections group, with a mean difference of −1.667, *p* < 0.001 under the equal-variance assumption and *p* = 0.001 under the unequal-variance assumption. The corresponding Cohen’s d was −0.312.

aPTT showed a borderline result under the equal-variance assumption and a significant result under the unequal-variance assumption. The mean aPTT was 26.006 ± 8.406 in the PE group and 24.781 ± 4.818 in the PE with associated infections group, with a mean difference of 1.2251. The two-sided *p*-value was 0.057 under the equal-variance assumption and 0.015 under the unequal-variance assumption. INR did not differ significantly between groups, with a mean difference of −0.02481 and a two-sided *p*-value of 0.398 under the equal-variance assumption.

### 3.5. Multivariable Logistic Regression for Death

Multivariable logistic regression analysis demonstrated that the PESI score and WBC count were independent predictors of in-hospital mortality (Table 4).

Tolerance and VIF values for all four predictors indicated no problematic multicollinearity (VIF < 5 for all variables; Table 4, footnote b); the modestly higher values for D-dimer and viral infection status (VIF = 2.07 for both) are consistent with their overlapping pathophysiology but did not approach a level of concern.

Each one-point increase in the PESI score increased the odds of death by 1.1% (OR = 1.011, 95% CI: 1.006–1.017, *p* < 0.001), while higher WBC values remained strongly associated with mortality (OR = 1.097, 95% CI: 1.060–1.135, *p* < 0.001).

Because our pre-specified primary model (Table 4) included only four of the variables that met the univariable significance threshold, we additionally report, in Table 5, a second model in which all such variables were entered simultaneously, to characterize their combined and independent contribution to mortality risk and to test whether the association between viral infection and mortality persists after adjustment for this broader covariate set.

In this full-variable model (*N* = 725, 150 events; Table 5), age (OR 1.028, *p* = 0.003), pulmonary hypertension (OR 0.529, *p* = 0.008), neutrophil count (OR 1.051, *p* < 0.001), GGT (OR 1.004, *p* = 0.015), procalcitonin (OR 1.120, *p* = 0.005), total cholesterol (OR 1.013, *p* < 0.001), Wells score (OR 1.136, *p* = 0.050), Padua score (OR 1.132, *p* = 0.011), and PESI score (OR 1.008, *p* = 0.043) were independently associated with in-hospital mortality. WBC, total bilirubin, AST, ALT, FBG, D-dimer, prothrombin time, INR, and IMPROVE score did not reach significance. Viral infection status showed the same direction of effect as in the primary model but did not reach statistical significance once the full covariate set was included (OR 1.890, 95% CI 0.931–3.835, *p* = 0.078).

**Figure 4:**
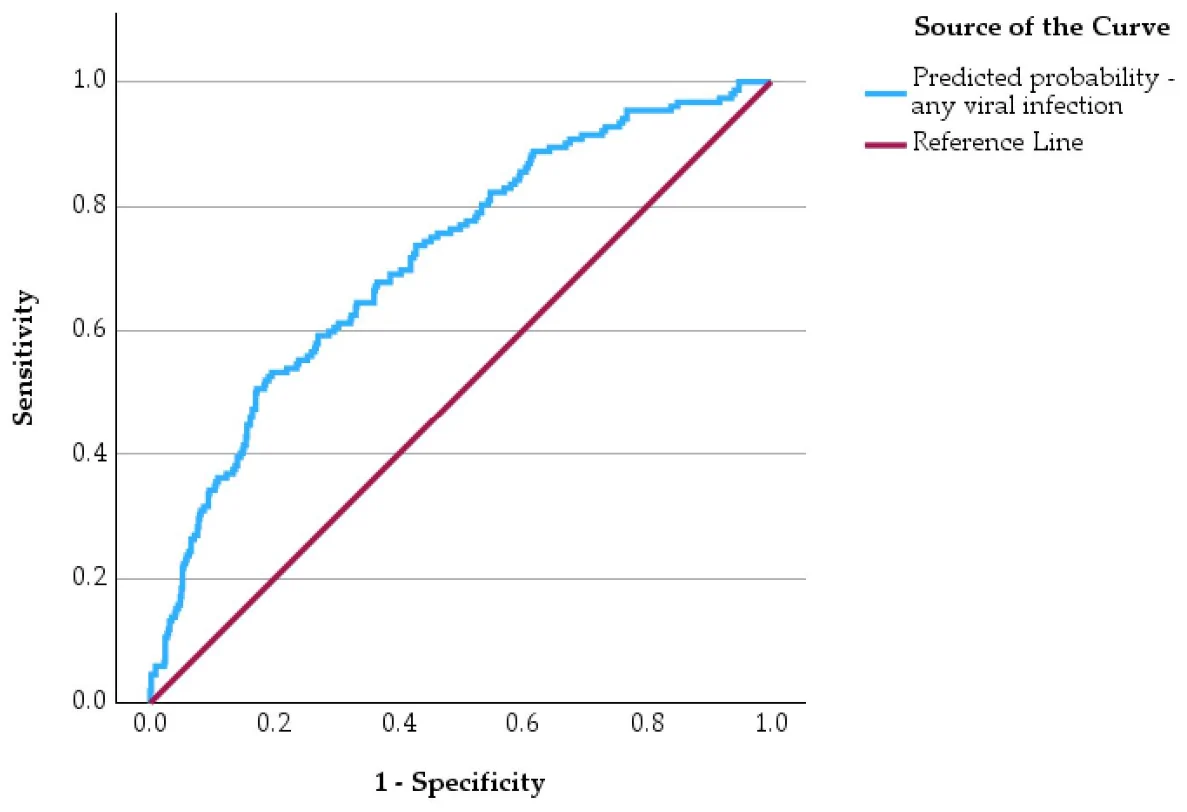
ROC curve for the combined model.

This multivariable logistic regression model presented in Table 4 demonstrated acceptable discriminatory performance for predicting in-hospital mortality (Figure 4). The combined model achieved an AUC of 0.715 (95% CI: 0.669–0.761, *p* < 0.001), indicating that integrating infection status with PESI score and WBC count improves mortality prediction beyond individual clinical risk scores.

The area under the ROC curve (AUC) was 0.715 (95% CI: 0.669–0.761, *p* < 0.001), indicating that the model had a statistically significant and fair ability to discriminate between patients who survived and those who died during hospitalization (Table 6).

The multivariable analysis demonstrated that the PESI score and WBC count were the most consistent independent predictors of in-hospital mortality.

**Figure 5:**
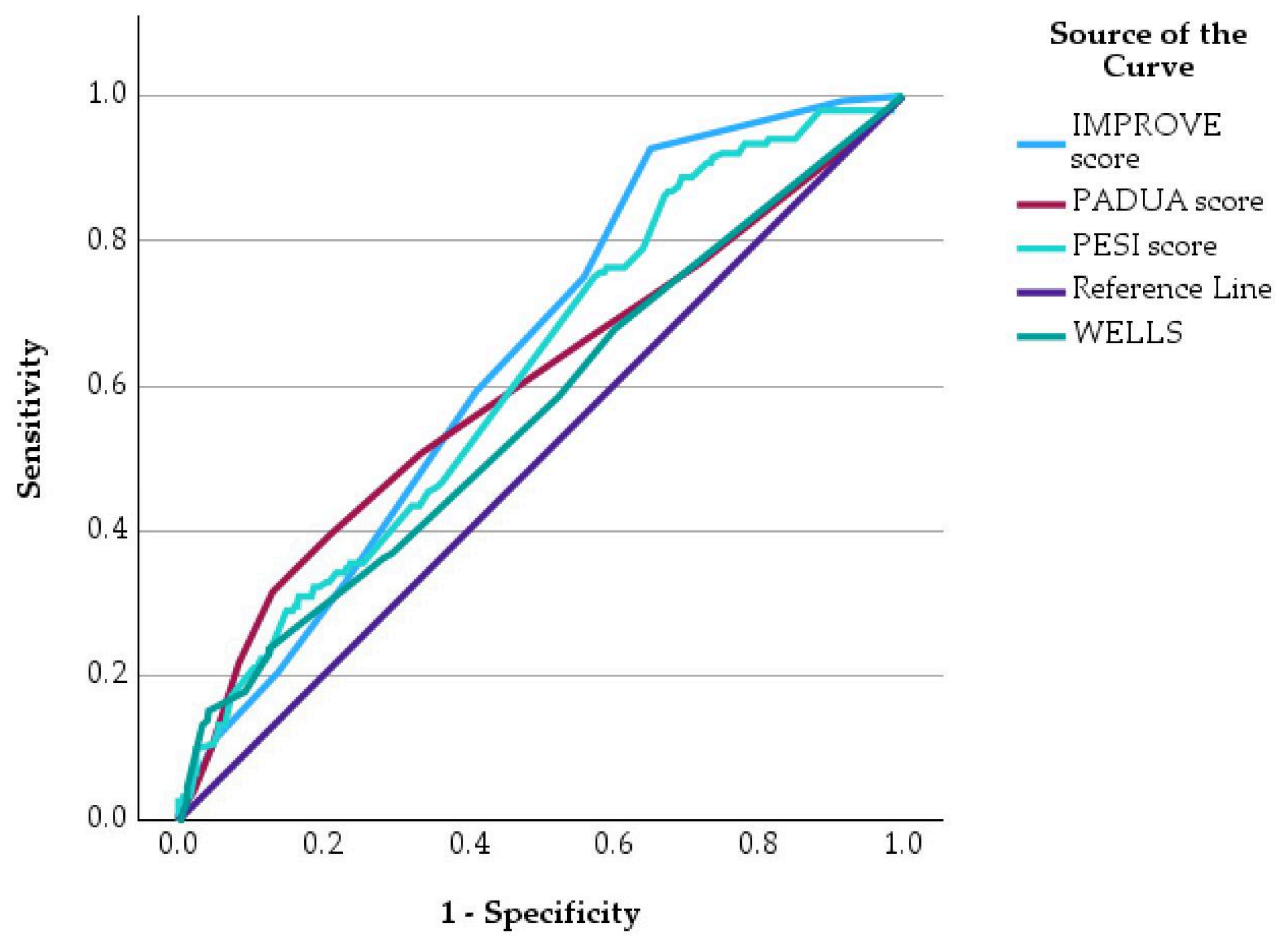
ROC curve for clinical risk scores.

ROC analysis demonstrated limited discriminatory performance of the individual clinical risk scores for predicting in-hospital mortality (Figure 5).

Among the evaluated scores, the IMPROVE score showed the highest discriminatory ability (AUC = 0.639, 95% CI: 0.595–0.684, *p* < 0.001), followed by the PESI score (AUC = 0.618, 95% CI: 0.569–0.666, *p* < 0.001), the Padua score (AUC = 0.598, 95% CI: 0.544–0.653, *p* < 0.001), and the Wells score (AUC = 0.562, 95% CI: 0.509–0.615, *p* = 0.022) (Table 7). Although all four scores performed significantly better than chance, their discriminatory ability remained poor (AUC < 0.70).

The multivariable analyses demonstrated that the PESI score and WBC count were the most consistent independent predictors of in-hospital mortality across both regression models. Importantly, the multivariable model demonstrated acceptable discriminatory performance (AUC > 0.70), outperforming the individual clinical risk scores, all of which exhibited poor discrimination (AUC < 0.70).

**Figure 6:**
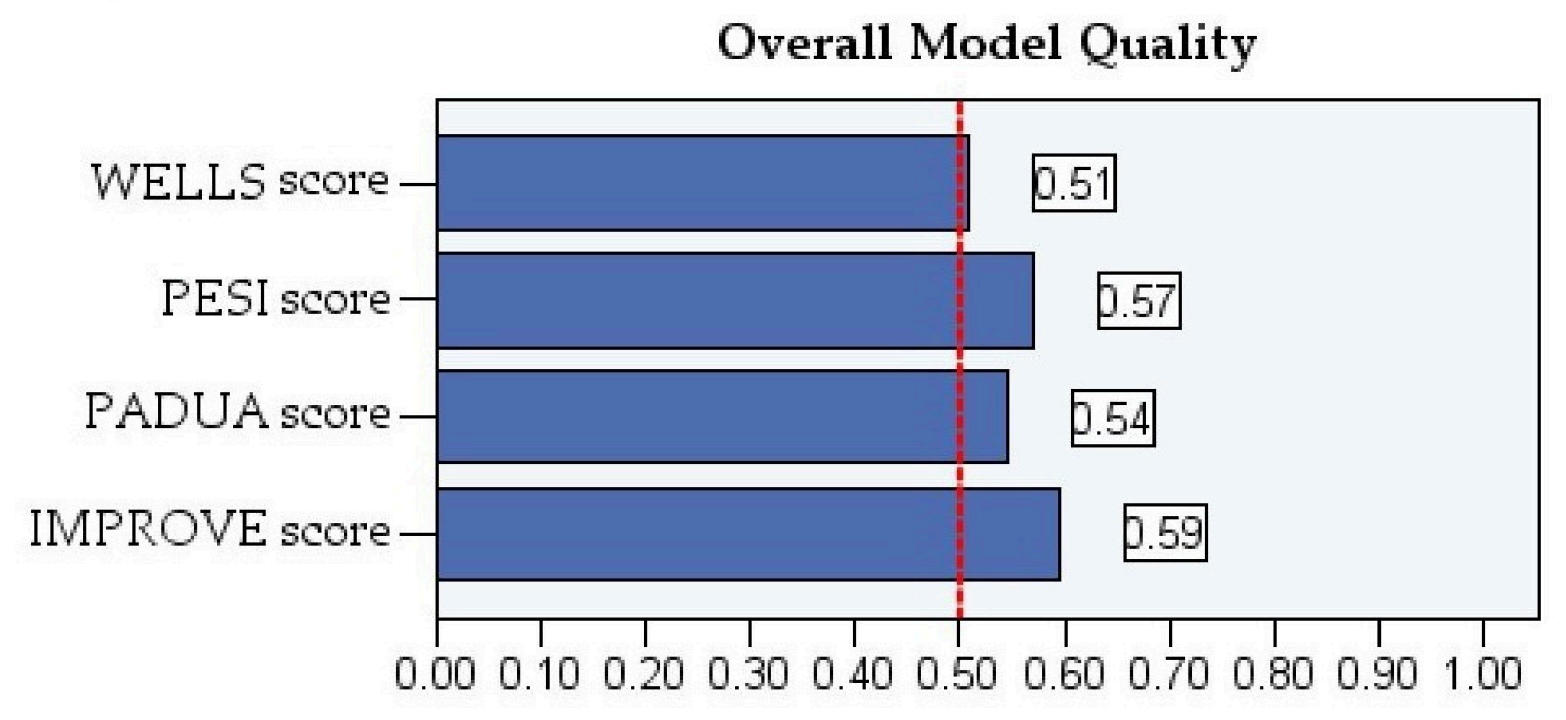
Overall discriminatory performance of the evaluated clinical risk scores based on ROC analysis.

The overall comparison of the evaluated clinical risk scores demonstrated modest discriminatory performance for predicting in-hospital mortality (Figure 6).

Among the assessed models, the IMPROVE score showed the highest overall performance, followed by the PESI score, whereas the Padua and Wells scores exhibited lower discriminatory ability. Although all models performed better than random classification, their predictive performance remained limited, supporting the subsequent development and evaluation of multivariable logistic regression models incorporating infection status and laboratory biomarkers to improve prognostic accuracy.

## 4. Discussion

Our findings support the clinical relevance of structured risk assessment in pulmonary embolism, but also show that infection-associated PE requires interpretation beyond standard score thresholds. In HOME-PE study group conducted by Roy et al. [11], Hestia criteria (a validated set of clinical criteria used to identify patients with PE eligible for outpatient management) and the sPESI strategies were safer for outpatient triage, with more than one-third of patients managed at home and low 30-day complication rates, whereas our cohort had higher crude mortality. The diuretic trial by Lim et al. [12] focused on normotensive intermediate-risk PE with sPESI ≥1 and right ventricular dysfunction, showing short-term clinical improvement without mortality separation, while our results emphasize infection burden, D-dimer elevation, and mortality prediction rather than acute hemodynamic intervention. Wong et al. [13] found Wells more specific than Revised Geneva for PE diagnosis, but our Wells ROC performance was weak for mortality; thus, diagnostic probability tools do not necessarily predict outcome after confirmed PE. Cohen et al. [14] reported that Padua combined with D-dimer improved PE exclusion in elderly patients, consistent with our observation that Padua alone had limited discrimination and may require biomarker integration. Similarly, oncology RAM studies show imperfect score performance. Foster et al. [15] found IMPROVE highly sensitive but nonspecific in hospitalized oncology patients, while Ratshabedi et al. [16] reported modest Padua performance in gynecologic cancer, paralleling our finding that IMPROVE had the highest AUC but limited independent predictive value. Our data suggest that PE prognosis in infected patients is best assessed through combined clinical, infectious, laboratory, and score-based evaluation.

Beyond established PE-specific severity scores, our results align with emerging literature describing infection-associated pulmonary embolism as a distinct clinical entity. The predominance of segmental artery involvement observed in our PE with associated infections group parallels imaging findings from COVID-19 cohorts, in which thrombi were more frequently peripheral (segmental/subsegmental), while the coexistence of infection and other comorbidities further emphasizes the need for individualized assessment of thromboembolic and bleeding risks when managing patients with PE [17]. The clinical relevance of infection during prolonged hospitalization is further underscored by evidence from medico-legal and hospital-based microbiological investigations showing that superimposed infections and antimicrobial-resistant organisms may complicate prolonged inpatient courses and contribute to diagnostic and post-mortem challenges [18]. Our finding of higher crude mortality and markedly longer hospital stay in the infection group is consistent with COVID-19-associated PE cohorts, where PE patients experienced longer hospitalization and higher in-hospital mortality compared with non-infected counterparts [19]. The elevated thrombotic risk specifically attributable to viral infection is further supported by large comparative cohort data showing that COVID-19 hospitalization carries a significantly higher 90-day VTE risk than influenza hospitalization [20], although both airborne respiratory viruses appear to activate overlapping prothrombotic pathways of endothelial injury, complement activation, and cytokine-driven coagulopathy [21]. Influenza-associated thromboembolism, though less extensively characterized than COVID-19, has similarly been linked to prolonged ICU stay, higher ventilatory support requirements, and increased mortality when thrombosis complicates severe infection, mirroring our observation that infection status was independently associated with death regardless of specific viral etiology [22,23,24].

Within each group, the length of stay was similar regardless of death outcome, suggesting that the prolonged hospitalization associated with infection was driven primarily by the infectious process itself rather than by proximity to a fatal outcome. The PE with associated infections group also showed wider interquartile ranges and higher upper extremes (up to 65–72 days) in both outcome categories.

Regarding risk-score performance, our finding that IMPROVE achieved the highest discriminatory ability among evaluated scores is consistent with external validation cohorts demonstrating good calibration of IMPROVE risk stratification in general medical inpatients [25], although head-to-head comparisons of venous thromboembolism risk-assessment models in unselected populations have similarly reported only modest overall accuracy [26], and incorporation of D-dimer has been shown to meaningfully improve IMPROVE’s risk discrimination [27,28]. Prior work demonstrating that biomarkers add incremental prognostic value beyond PESI alone further supports our multivariable finding that WBC count, an inflammatory biomarker, independently improved mortality prediction beyond PESI [29]. This parallels ICU-based PE cohorts, where indices with additional physiological markers improved discrimination for hospital survival beyond PESI alone [30].

This study did not include a comparison with contemporary PE-specific severity classifications, such as the 2019 ESC pulmonary embolism risk classification or the current AHA pulmonary embolism risk classification, as these require hemodynamic, right-ventricular-function, and biomarker data not uniformly available for retrospective reconstruction. Future prospective studies incorporating these classifications alongside PESI, Wells, Padua, and IMPROVE would further clarify their comparative clinical utility.

Because COVID-19 accounted for 172/191 (90.1%) of infections in the PE with associated infections group, our findings predominantly characterize COVID-19-associated pulmonary embolism rather than viral respiratory infections in general. This distribution reflects the fact that patient recruitment occurred during the COVID-19 pandemic, a period in which COVID-19 was the dominant circulating respiratory viral pathogen. COVID-19 is itself a well-established independent risk factor for venous thromboembolism, given its association with endothelial injury, hypercoagulability, and a pronounced systemic inflammatory response; its predominance in our cohort therefore reflects the epidemiological context of the study period rather than a selection bias. Accordingly, results should not be overgeneralized to other viral etiologies.

The higher prevalence of pulmonary hypertension among survivors (43.4%) than non-survivors (25.7%, *p* < 0.001) is counterintuitive and warrants further comment. We consider several non-mutually exclusive explanations. First, pulmonary hypertension in this cohort was recorded as a pre-existing, chronically diagnosed comorbidity rather than as an acute hemodynamic complication of the index PE; patients with a known chronic diagnosis may already have been established on directed therapy and under closer specialist follow-up, potentially prompting earlier PE recognition and treatment. Second, ascertainment bias may contribute: a pre-existing cardiopulmonary diagnosis increases the likelihood of closer hemodynamic monitoring and imaging surveillance, whereas patients who died rapidly after a severe acute PE may not have had a pre-existing diagnosis established or documented in the available records. Third, residual confounding by indication cannot be excluded, since comorbidity burden and PE severity are not perfectly correlated. Finally, this finding may reflect survival/selection bias inherent to a retrospective cohort: patients who die acutely from severe PE are, by definition, unable to accrue the additional clinical encounters through which a chronic pulmonary hypertension diagnosis is typically established, which would understate its true prevalence among non-survivors. Pulmonary hypertension was not among the four pre-specified variables in our primary model (Table 4), but it did remain an independent, protective predictor of in-hospital mortality in the full-variable model (Table 5; OR 0.529, 95% CI 0.329–0.849, *p* = 0.008), in the same direction as its univariable association. Given the cross-sectional, non-causal way pulmonary hypertension was ascertained in this cohort, this association should still be interpreted with caution, and prospective studies with pre-PE pulmonary hypertension ascertainment are needed to clarify this relationship.

A limitation of the thrombus-localization data is that bilateral involvement and saddle-embolus configuration were not captured as distinct, separately coded variables; these presentations are included within the ‘other localizations’ category, which precludes a granular breakdown of thrombus distribution and morphology independent of anatomical level.

Also, although this study was restricted to laboratory-confirmed viral infections, bacterial infections are independently associated with an elevated risk of venous thromboembolism through overlapping mechanisms of endothelial injury, platelet activation, and cytokine-driven coagulopathy [31,32,33]. Septic pulmonary embolism arising from bacteremic sources such as Staphylococcus aureus represents a further, mechanistically distinct entity associated with substantial morbidity and mortality [34]. The broader clinical management of patients with severe or fatal disease may also have important medico-legal implications, particularly when accurate documentation and identification are required in hospital and post-mortem settings [35]. Because our cohort was defined exclusively by viral infection status, these findings cannot be extrapolated to PE associated with bacterial infection, and dedicated studies comparing viral and bacterial infection-associated PE phenotypes are warranted.

In the full-variable model that simultaneously entered all 18 candidate covariates reaching *p* < 0.05 in univariable comparisons (Table 5), this association weakened to non-significance (OR 1.890, 95% CI 0.931–3.835, *p* = 0.078), while age, pulmonary hypertension, neutrophil count, GGT, procalcitonin, cholesterol, Wells score, Padua score, and PESI score emerged as independent predictors. This pattern suggests that part of the excess mortality observed in virally infected PE patients may be mediated through inflammatory (neutrophilia, procalcitonin), hepatic, and metabolic pathways captured by these additional variables, rather than reflecting a fully independent, infection-specific mechanism, though the viral infection estimate retained the same direction of effect as in the primary model and its confidence interval remained compatible with a clinically meaningful association. We present both models transparently: the pre-specified four-variable model (Table 4) as our primary analysis, addressing our a priori hypothesis regarding viral infection, and the full-variable model (Table 5) as a complementary analysis exploring the broader clinical picture. Future studies with formal mediation analysis and larger infected-subgroup sample sizes are needed to disentangle these overlapping pathways.

## 5. Conclusions

Associated infections appear to define a distinct and clinically more severe phenotype among patients with pulmonary embolism. In this cohort, patients with infection-associated PE had higher crude mortality, increased PESI and IMPROVE scores, greater inflammatory and coagulation abnormalities, and a different thrombus distribution compared with patients without associated infections. Among the evaluated risk scores, IMPROVE showed the highest univariable ROC discrimination, whereas only the PESI score remained independently associated with mortality in multivariable analysis.

Conventional PE and VTE scores provide useful but incomplete prognostic information when infection is present. Infection type, systemic inflammatory response, D-dimer elevation, and coagulation changes should therefore be integrated with established scoring systems to improve risk stratification. Future prospective studies are needed to validate combined models incorporating PE-specific scores, infection-related variables, and laboratory markers for outcome prediction in this high-risk population.

## Figures and Tables

**Table 1:** Baseline characteristics by in-hospital outcome.

Variable	Survivors (*n* = 577)	Non-Survivors (*n* = 152)	Test Statistic	*p*
Age, years	67.99 ± 13.95	71.39 ± 13.96	t = −2.669	0.008
Sex, male—*n* (%)	301/577 (52.2%)	71/152 (46.7%)	χ^2^ = 1.223	0.269
Comorbidities—*n* (%)				
Pulmonary hypertension	250/576 (43.4%)	39/152 (25.7%)	χ^2^ = 15.087	<0.001
Diabetes	144/576 (25.0%)	33/152 (21.7%)	χ^2^ = 0.540	0.463
Cancer	104/577 (18.0%)	31/152 (20.4%)	χ^2^ = 0.305	0.581
Cardiac failure	383/576 (66.5%)	97/152 (63.8%)	χ^2^ = 0.274	0.601
Thrombus localization—*n* (%)				
Main pulmonary artery	131/576 (22.7%)	27/151 (17.9%)		
Lobar artery	164/576 (28.5%)	36/151 (23.8%)		
Segmental artery	174/576 (30.2%)	65/151 (43.0%)		
Subsegmental artery	18/576 (3.1%)	2/151 (1.3%)		
Truncus pulmonalis	18/576 (3.1%)	4/151 (2.6%)		
Other localisations	71/576 (12.3%)	17/151 (11.3%)		
Overall comparison (6 categories)			χ^2^ = 9.844, df = 5	0.080
Laboratory parameters, mean ± SD				
WBC, ×10^3^/µL	9.17 ± 4.81	13.28 ± 10.51	U = 29,919.0	<0.001
Neutrophils, ×10^3^/µL (*n* = 576/152)	7.99 ± 5.95	15.98 ± 19.46	U = 28,582.5	<0.001
Lymphocytes, ×10^3^/µL	3.55 ± 3.90	4.13 ± 5.67	U = 46,463.0	0.258
Platelet count, ×10^3^/µL	232.48 ± 94.28	225.40 ± 126.74	t = 0.643	0.521
Creatinine, mg/dL (*n* = 577/151)	1.31 ± 3.93	1.17 ± 0.59	U = 44,317.0	0.743
Total bilirubin, mg/dL (*n* = 577/151)	0.72 ± 0.76	0.89 ± 0.96	U = 36,649.0	0.003
AST, U/L (*n* = 577/151)	58.91 ± 194.46	82.38 ± 226.70	U = 33,917.5	<0.001
ALT, U/L (*n* = 577/151)	61.81 ± 191.59	83.05 ± 200.30	U = 37,031.5	0.004
GGT, U/L (*n* = 577/151)	67.02 ± 58.10	94.08 ± 88.57	U = 34,287.5	<0.001
Glucose, mg/dL (*n* = 577/151)	142.68 ± 66.70	156.64 ± 73.22	U = 36,612.5	0.003
Sodium, mmol/L (*n* = 577/151)	137.68 ± 4.83	138.48 ± 6.02	t = −1.525	0.129
Potassium, mmol/L (*n* = 577/151)	4.10 ± 0.57	4.14 ± 0.69	t = −0.598	0.550
Procalcitonin, ng/mL (*n* = 577/150)	0.84 ± 2.03	2.14 ± 5.74	U = 30,859.0	<0.001
Total cholesterol, mg/dL	166.67 ± 45.61	199.94 ± 65.59	t = −5.890	<0.001
D-dimers, µg/mL FEU	14.48 ± 18.24	20.01 ± 22.33	U = 50,078	<0.007
Prothrombin time, s (*n* = 577/150)	14.58 ± 5.78	15.28 ± 3.51	U = 54,525.0	<0.001
aPTT, s (*n* = 577/150)	25.77 ± 7.90	25.35 ± 6.61	U = 42,099.5	0.448
INR (*n* = 577/152)	1.16 ± 0.36	1.21 ± 0.28	U = 51,244.5	<0.001
Clinical risk scores, mean ± SD				
PESI score	110.29 ± 32.26	125.76 ± 35.88	t = −4.826	<0.001
Wells score	1.54 ± 1.73	2.11 ± 2.21	U = 38,395.5	0.014
Padua score	3.03 ± 2.12	3.95 ± 2.61	t = −4.004	<0.001
IMPROVE score	2.98 ± 2.07	3.97 ± 1.87	t = −5.641	<0.001
Infection status—*n* (%)				
Any viral infection	138/577 (23.9%)	53/152 (34.9%)	χ^2^ = 6.907	0.009

Abbreviations: ALT, alanine aminotransferase; aPTT, activated partial thromboplastin time; AST, aspartate aminotransferase; GGT, gamma-glutamyl transferase; INR, international normalized ratio; PESI, Pulmonary Embolism Severity Index; SD, standard deviation; WBC, white blood cell count. **Statistical tests:** Continuous variables were compared using the independent-samples *t*-test (t) for approximately normally distributed variables (|skewness| ≤ 1 in both groups) or the Mann–Whitney U test (U) otherwise. Categorical variables were compared using the Pearson chi-square test (χ^2^).

**Table 2:** Baseline characteristics, comorbidities, thrombus location, risk-stratification scores, and clinical outcome according to study group.

Variable	PE (*n* = 538)					PE with Associated Infections (*n* = 191)					*p*-Value
	*N*	%	Mean	SD	Median	*N*	%	Mean	SD	Median	
Demographic characteristics											
Sex—Male	265	49.3				107	56				0.108
Sex—Female	273	50.7				84	44				
Thrombus location											
Main artery	138	25.7				20	10.5				
Lobar artery	159	29.7				41	21.5				
Segmental artery	156	29.1				83	43.5				
Subsegmental artery	20	3.7				0	0				
Truncus pulmonalis	21	3.9				1	0.5				
Other localizations	42	7.8				46	24.1				
Risk-stratification scores											
PESI class			3	1	4			4	1	4	
PESI score			110	32.9	110			123.41	33.71	116	
WELLS score			1.83	1.95	1.5			1.16	1.45	1	
PADUA score			3.34	2.34	3			2.89	2.02	3	
IMPROVE score			2.62	1.88	2			4.8	1.68	5	
Comorbidities											
Pulmonary hypertension—No	304	56.5				135	71.1				<0.001
Pulmonary hypertension—Yes	234	43.5				55	28.9				
Diabetes—No	415	77.1				136	71.6				0.125
Diabetes—Yes	123	22.9				54	28.4				
Cancer—No	431	80.1				163	85.3				0.11
Cancer—Yes	107	19.9				28	14.7				
Obesity—No	370	68.8				117	61.6				0.085
Obesity—Yes	168	31.2				73	38.4				
Cardiac failure—No	184	34.2				64	33.7				0.897
Cardiac failure—Yes	354	65.8				126	66.3				
Viral infection status											
No infection	538	100				0	0				<0.001
COVID-19	0	0				172	90.1				
Influenza A	0	0				4	2.1				
Influenza B	0	0				7	3.7				
Respiratory syncytial virus	0	0				5	2.6				
Human metapneumovirus	0	0				2	1				
Parainfluenza	0	0				1	0.5				
Clinical outcome											
In-hospital death—No	439	81.6				138	72.3				0.006
In-hospital death—Yes	99	18.4				53	27.7				

**Table 3:** Laboratory profile and risk-stratification scores, with between-group comparisons, according to study group.

Variable	PE (*n* = 538) Mean ± SD	PE Median	PE + Infection (*n* = 191) Mean ± SD	PE + Infection Median	Mean Difference ^1^	*p*-Value	Cohen’s d
Demographic characteristics and risk-stratification scores							
Age, years	68.55 ± 14.22	70	69.12 ± 13.43	71	−0.568	0.630/0.621 ^2^	−0.041
PESI score	110.00 ± 32.90	110.00	123.41 ± 33.71	116.00	−13.406	<0.001	−0.405
WELLS score	1.83 ± 1.95	1.50	1.16 ± 1.45	1.00	0.670	<0.001	0.365
PADUA score	3.34 ± 2.34	3.00	2.89 ± 2.02	3.00	0.446	0.019/0.012 ^2^	0.198
IMPROVE score	2.62 ± 1.88	2.00	4.80 ± 1.68	5.00	−2.179	<0.001	−1.189
Laboratory profile							
WBC (×10^3^/µL)	9.88 ± 5.49	10	12.21 ± 5.61	11	−2.330	<0.001	−0.422
Neutrophils (×10^3^/µL)	10 ± 12	8	8 ± 5	7	—	0.018	—
Lymphocytes (×10^3^/µL)	2 ± 2	2	9 ± 5	8	—	<0.001	—
PLT (×10^3^/µL)	231 ± 97	217	230 ± 115	215	—	0.844	—
Creatinine (mg/dL)	1.3 ± 4.0	1.0	1.0 ± 0.5	0.9	—	<0.001	—
Total bilirubin (mg/dL)	0.7 ± 0.7	0.6	0.7 ± 0.8	0.5	—	0.053	—
AST (U/L)	67 ± 233	31	53 ± 38	44	—	<0.001	—
ALT (U/L)	67 ± 223	32	65 ± 50	56	—	<0.001	—
GGT (U/L)	71 ± 72	49	77 ± 45	66	—	<0.001	—
FBG (mg/dL)	146 ± 70	126	144 ± 62	123	—	0.678	—
Na (mmol/L)	139 ± 5	139	136 ± 4	135	—	<0.001	—
K (mmol/L)	4.1 ± 0.6	4.1	4.1 ± 0.6	4.0	—	0.427	—
Procalcitonin (ng/mL)	1.1 ± 3.5	0.2	0.9 ± 1.7	0.3	—	<0.001	—
Cholesterol (mg/dL)	165 ± 50	160	198 ± 50	187	—	<0.001	—
D-dimers (µg/mL FEU)	8.79 ± 10.97	5	34.91 ± 24.05	27	−26.111	<0.001	−1.686
Prothrombin time (s)	14.29 ± 4.86	13	15.96 ± 6.51	14	−1.667	<0.001/0.001 ^2^	−0.312
aPTT (s)	26.006 ± 8.406	24.6	24.781 ± 4.818	23.9	1.225	0.057/0.015 ^2^	0.161
INR	1.1604 ± 0.3269	1.1	1.1852 ± 0.4021	1.1	−0.025	0.398	−0.071

WBC, white blood cell count; PLT, platelet count; AST, aspartate aminotransferase; ALT, alanine aminotransferase; GGT, gamma-glutamyl transferase; FBG, fasting blood glucose; Na, sodium; K, potassium; INR, international normalized ratio; SD, standard deviation. ^1^ Mean difference calculated as PE minus PE with associated infections. ^2^ Two values given where the equal-variance and unequal-variance (Welch) assumptions yielded different *p*-values, in the order equal-variance/unequal-variance. —, mean difference and Cohen’s d not computed in the original independent-samples analysis.

**Table 4:** Multivariable logistic regression model for in-hospital mortality (combined model).

		B	S.E.	Wald	df	Sig.	Exp (B)	95% C.I. for EXP (B)	
								Lower	Upper
Step 1 ^a^	PESI score	0.011	0.003	14.889	1	<0.001	1.011	1.006	1.017
	WBC	0.093	0.017	28.227	1	<0.001	1.097	1.060	1.135
	D dimers	0.001	0.005	0.028	1	0.866	1.001	0.991	1.011
	Any viral infection	0.639	0.204	9.760	1	0.002	1.894	1.269	2.828
	Constant	−4.046	0.416	94.459	1	<0.001	0.017		

^a^ Variable(s) entered on step 1: PESI score, WBC, D dimers, Any viral infection.

**Table 5:** Multivariable logistic regression analysis of predictors of in-hospital mortality.

		B	S.E.	Wald	df	Sig.	Exp (B)	95% C.I. for EXP (B)	
								Lower	Upper
Step 1 ^a^	Age	0.028	0.009	8.637	1	0.003	1.028	1.009	1.047
	Pulmonary hypertension	−0.637	0.242	6.944	1	0.008	0.529	0.329	0.849
	WBC	0.037	0.021	2.905	1	0.088	1.037	0.995	1.082
	Neutrophils	0.049	0.014	12.426	1	<0.001	1.051	1.022	1.080
	Total bilirubin	0.173	0.106	2.700	1	0.100	1.189	0.967	1.463
	AST	0.002	0.002	0.536	1	0.464	1.002	0.997	1.006
	ALT	−0.001	0.002	0.296	1	0.586	0.999	0.994	1.003
	GGT	0.004	0.002	5.869	1	0.015	1.004	1.001	1.007
	FBG	−0.001	0.002	0.337	1	0.561	0.999	0.996	1.002
	Procalcitonin	0.113	0.040	7.967	1	0.005	1.120	1.035	1.212
	Cholesterol	0.013	0.002	29.252	1	<0.001	1.013	1.009	1.018
	D dimers	0.001	0.006	0.024	1	0.876	1.001	0.990	1.012
	Prothrombin time	0.014	0.028	0.239	1	0.625	1.014	0.960	1.071
	INR	0.195	0.372	0.275	1	0.600	1.216	0.586	2.520
	WELLS score	0.127	0.065	3.833	1	0.050	1.136	1.000	1.290
	PADUA score	0.124	0.049	6.435	1	0.011	1.132	1.029	1.246
	IMPROVE score	−0.069	0.081	0.737	1	0.391	0.933	0.796	1.093
	PESI score	0.008	0.004	4.109	1	0.043	1.008	1.000	1.016
	Any viral infection	0.636	0.361	3.106	1	0.078	1.890	0.931	3.835
	Constant	−9.033	1.014	79.297	1	<0.001	0.000		

^a^ Variable(s) entered on step 1: Age, Pulmonary hypertension, WBC, Neutrophils, Total bilirubin, AST, ALT, GGT, FBG, Procalcitonin, Cholesterol, D dimers, Prothrombin time, INR, WELLS, PADUA score, IMPROVE for VTE, Pessi score, Any viral infection.

**Table 6:** Area Under the ROC Curve for the combined model.

Test Result Variable (s): Predicted Probability of the Combined Model				
Area	Std. Error ^a^	Asymptotic Sig. ^b^	Asymptotic 95% Confidence Interval	
			Lower Bound	Upper Bound
0.715	0.023	0.000	0.669	0.761

^a^ Under the nonparametric assumption. ^b^ Null hypothesis: true area = 0.5.

**Table 7:** Area Under the ROC Curve for clinical risk scores.

Test Result Variable (s)	Area	Std. Error ^a^	Asymptotic Sig. ^b^	Asymptotic 95% Confidence Interval	
				Lower Bound	Upper Bound
PESI score	0.618	0.025	0.000	0.569	0.666
Wells score	0.562	0.027	0.022	0.509	0.615
PADUA score	0.598	0.028	0.000	0.544	0.653
IMPROVE score	0.639	0.023	0.000	0.595	0.684

The test result variable(s): PESI score, Wells score, Padua score, IMPROVE score has at least one tie between the positive actual state group and the negative actual state group. ^a^ Under the nonparametric assumption. ^b^ Null hypothesis: true area = 0.5.

## Data Availability

Data is available on request to the corresponding author due to ethical reasons.

## Abbreviations

The following abbreviations are used in this manuscript:

ALT	Alanine aminotransferase
aPTT	Activated partial thromboplastin time
AST	Aspartate aminotransferase
AUC	Area under the curve
COVID-19	Coronavirus disease 2019
GGT	Gamma-glutamyl transferase
INR	International normalized ratio
IMPROVE	International Medical Prevention Registry on Venous Thromboembolism
OR	Odds ratio
PE	Pulmonary embolism
PESI	Pulmonary Embolism Severity Index
PH	Pulmonary hypertension
PLT	Platelet count
ROC	Receiver operating characteristic
RSV	Respiratory syncytial virus
SD	Standard deviation
VTE	Venous thromboembolism
WBC	White blood cell count
